# The Physical and Psychological Impact of Multiple Sclerosis Using the MSIS-29 via the Web Portal of the UK MS Register

**DOI:** 10.1371/journal.pone.0055422

**Published:** 2013-01-31

**Authors:** Kerina H. Jones, David V. Ford, Philip A. Jones, Ann John, Rodden M. Middleton, Hazel Lockhart-Jones, Jeffrey Peng, Lisa A. Osborne, J. Gareth Noble

**Affiliations:** 1 College of Medicine, Swansea University, Swansea, Wales, United Kingdom; 2 Long Term and Chronic Conditions Centre, College of Human and Health Sciences, Swansea University, Swansea, Wales, United Kingdom; Innsbruck Medical University, Austria

## Abstract

**Introduction:**

The MSIS-29 was developed to assess the physical and psychological impact of MS. The aims of this study were to use the responses to the MSIS-29 via the web portal of the UK MS Register to: examine the internal properties of the scale delivered via the internet, profile the cohort, and assess how well the scale measures impact of disability on the potential workforce.

**Methods:**

Between May 2011 and April 2012, 4558 people with MS completed the MSIS-29(v.1). The responses were collated with basic demographic and descriptive MS data and the resulting dataset was analysed in SPSS(v.20).

**Results:**

Internal consistency was high (Cronbach's alpha 0.97 MSIS-29-PHYS, 0.92 MSIS-29-PSYCH). The mean MSIS-29-PHYS score was 60.5 (50.6%) with a median of 62 and the mean MSIS-29-PSYCH score was 24.8 (43.8%) with a median of 24. Physical scores increased with age and disease duration (*p*<0.001, *p*<0.001), but there was a weak negative relationship between psychological scores and age (*p*<0.001). The odds of people having an employment status of sick/disabled were 7.2 (CI 5.5, 9.4, *p*<0.001) for people with a moderate physical score, and 22.3 (CI 17.0, 29.3, *p*<0.001) for people with a high physical score (relative to having a low physical score).

**Conclusions:**

This largest known study of its kind has demonstrated how the MSIS-29 can be administered via the internet to characterise a cohort, and to predict the likely impact of disability on taking an active part in the workforce, as a reasonable proxy for the effects of MS on general activities. The findings examining MSIS-29-PHYS and MSIS-29-PSYCH scores against age support the use of two sub-scales, not a combined score. These results underline the importance of using a scale such as this to monitor disability levels regularly in guiding MS care to enable people to be as active as possible.

## Introduction

Multiple Sclerosis (MS) is a chronic, inflammatory, autoimmune condition, well-known for resulting in physical and cognitive disabilities over the disease course. It is important to be able to assess the impact of any chronic disease and often general tools are used (such as the Hospital Anxiety and Depression Scale (HADS) [1] in conjunction with instruments designed for the disease type. The Multiple Sclerosis Impact Scale (MSIS-29) was developed as such a disease-specific instrument to assess the physical and psychological impact of living with MS [2]. It consists of 29 questions divided into two components: a physical scale (MSIS-29-PHYS) of 20 questions, and a psychological scale (MSIS-29-PSYCH) of 9 questions. The responses are scored on a Likert scale (1–5, version 1) and summed to give a maximum of 100 on MSIS-29-PHYS and 45 on MSIS-29-PSYCH. These raw scores can be transformed to create two scales each spanning 0 to 100 and expressed as percentages [2], [3]. The MSIS-29 scale has been subjected to rigorous evaluations to demonstrate its validity, reliability and psychometric properties [2]–[4], its suitability for assessing people with MS in comparison with other established measures, and for proxy use with partners of people with MS [5]–[9]. As a result, it is reported to be a valid and reliable method of assessing quality of life and is increasingly being used in MS research and clinical settings [3]–[5], [10].

The UK MS Register aims to address the need for an increased knowledge-base about MS by bringing together datasets from multiple sources to create a rich resource for research, policy development and service planning. It has been designed to capture data from three main sources, with the ability to anonymously link these data at the individual level whilst retaining privacy [11], [12]. Data are collected directly from people with MS via a purpose-built web portal, from sources of routine administrative data, and from clinical information systems operating in NHS neurology clinics. The web portal was launched in May 2011 and, by April 2012, 8736 people with MS had enrolled, with further data collection underway as a continual process. Data acquisition from clinics and administrative sources is underway, subject to consent and data availability. In bringing together data from three disparate sources the UK MS Register model is innovative in its design and provides new opportunities for studying MS via linked data. The Register is based on the proven technologies and robust Information Governance arrangements in place in the Secure Anonymised Information Linkage (SAIL) system developed by the Health Information Research Unit (HIRU) [13], [14].

The web portal acts as a questionnaire delivery platform so that people with MS can provide information on their experiences of living with MS. It hosts a number of validated questionnaires, including: the Hospital Anxiety and Depression Scale (HADS) [1], the EQ-5D [15] and the MS Disease Impact Scale-29 (MSIS-29) [2]. These cover a range of topics such as: MS and mental well-being; impact of MS on daily life; lifestyle and health outcomes. Baseline information including: age, gender, date of diagnosis of MS, type of MS, age at onset and employment status is collected as part of the registration process and a questionnaire entitled ‘You, your MS and lifestyle’ [11]. We have shown that it is feasible to collect information in this way and to use it to characterize a cohort of people with MS, and to study the mental well-being of people with MS via the HADS [11], [16]. Responses gained via the web portal provided the opportunity for the largest known study of the physical and psychological impacts of MS using the MSIS-29.

### Research aim

In their development and validation work on the MSIS-29, Hobart and colleagues [2], [3] recommended that the scale should be used in different settings, and so we sought to examine its internal properties when delivered via the internet and to compare them with published findings. We then set out to profile and characterise our cohort of people with MS using descriptive statistics. There were two main reasons for this: firstly, this is the first paper from the UK MS Register that uses the MSIS-29 responses, and we wanted to describe this cohort for researchers who may wish to use Register data in the future; and, secondly, since it is a large cohort we believe the findings will be of interest for comparative purposes with other studies that may not have access to such large numbers. Furthermore, since the MSIS-29 is designed to measure disability, and we collect data on the employment status of people with MS, we sought to find out the extent to which the scores translate into, and predict, an employment status of sick/disabled. This would show how well the scale can be used to measure the impact of disability on the potential workforce, which may also act as a reasonable proxy for impact on general life activities.

## Methods

### Research ethics and governance

The UK MS Register study was peer-reviewed via the MS Society and it received ethical approval from the South West – Central Bristol Research Ethics Committee (11/SW/0160) as a research database [17]. Under this ethical approval, data collected via the portal, the neurology clinics and routine administrative sources can be anonymously linked using the SAIL methodologies provided that agreement to the portal terms of service (via the portal) and written informed participant consent (at the clinics) have been obtained. The working UK MS Register contains only anonymous data but facilities are in place to re-contact participants to take part in further research [18]. In future, the Register data will be made accessible for analysis by researchers external to the team, subject to regulatory and governance requirements. The final operating model for these arrangements is yet to be determined, but we are able at accommodate researcher requests to view the data in the interim, subject to any necessary amendments to regulatory and governance approvals and a non-disclosure agreement.

### Data collection and analysis

Adults with MS living in the UK have been able to enrol on the web portal of the UK MS Register since its launch in May 2011. Up to April 2012 we received 4558 complete responses to the MSIS-29 (version 1) via the web portal. These were collated with basic demographic, descriptive MS data and employment status and the resulting dataset was analysed in SPSS (v.20). Raw scores (physical: 20 to 100, psychological: 9 to 45) and transformed scores (%) were both used for ease of reference, and the physical (MSIS-29-PHYS) and psychological (MSIS-29-PSYCH) scores were treated as separate sub-scales in all the analyses in accordance with the suggested guidance of Hobart and colleagues [2], [3]. Descriptive statistics were used to characterize the cohort of people with MS. Cronbach's alpha was used to assess the internal consistency of MSIS-29-PHYS and MSIS-29-PSYCH. Frequencies were studied by categorising the physical and psychological scales into tertiles with thresholds of 33.3%, 66.7% and 100% to represent low, moderate and high scores, respectively, and chi squared tests were used to assess goodness of fit. The continuous variables were assessed for normality using the Kolmogorov-Smirnov test and all were found to deviate significantly from the normal distribution (*p*<0.001). Because of this, non-parametric inferential tests were used: Spearman's rank correlation coefficient to measure relationships between variables, the Mann-Whitney U test was used to assess differences between two independent samples, and the Kruskal-Wallis one-way analysis of variance was used to compare more than two independent samples. Logistic regression was used to predict the outcome of categorical variables using the tertiles for MSIS-29-PHYS and MSIS-29-PSYCH, and the raw MSIS-29-PHYS scores in bands of 10 (above the minimum score of 20).

## Results

### Internal consistency and correlation

The properties of the MSIS-29 have been demonstrated, and we have been able to show similarly good results using our data obtained via the web portal [2]–[5], [7] The internal consistency of the MSIS-29-PHYS and MSIS-29-PSYCH question scores were assessed using Cronbach's alpha and were found to be 0.97 and 0.92 respectively. There was a fairly strong positive relationship between the MSIS-29-PHYS and MSIS-29-PSYCH scores (*rho* = 0.60, *p*<0.001). The mean MSIS-29-PHYS and MSIS-29-PSYCH scores were around the middle of the scales (Table 1) and the floor and ceiling effects were negligible (1.3% of respondents were at scale minimum and 1.2% at scale maximum for MSIS-29-PHYS, 1.6% were at scale minimum and 1.0% at scale maximum for MSIS-29-PSYCH).

**Table 1:** Timelines and MSIS-29 scores of the cohort.

Categories	N	Mean	SD	SE	Median	IQR	Range
**Age (yrs):**							
All	4547	50.7	11.2	0.17	51.0	16	20 to 87
Male	1310	52.8	11.4	0.31	53.0	17	23 to 87
Female	3229	50.5	11.4	0.20	50.0	16	20 to 84
**Time from first symptoms (yrs):**							
All	3624	16.3	11.2	0.19	14.0	16	0 to 63
Male	1039	16.5	11.3	0.35	14.0	16	0 to 62
Female	2567	16.2	11.2	0.22	14.0	16	0 to 63
**Time since diagnosis (yrs):**							
All	3606	10.9	8.9	0.15	9.0	12	0 to 64
Male	1031	11.3	8.9	0.28	10.0	13	0 to 48
Female	2557	10.8	8.9	0.18	9.0	12	0 to 64
**MSIS-29-PHYS score (raw):**							
All	4558	60.5	21.6	0.32	62.0	35	20 to 100
PPMS	659	70.0	17.9	0.70	72.0	27	22 to 100
RRMS	2769	56.7	21.9	0.42	57.0	37	20 to 100
SPMS	362	73.6	16.0	0.84	74.0	22	28 to 100
DKMS	672	59.3	21.2	0.82	61.5	36	20 to 100
**MSIS-29-PHYS score (%):**							
All	4558	50.6	27.0	0.40	52.5	44	0 to 100
PPMS	659	62.5	22.4	0.87	65.0	34	3 to 100
RRMS	2769	45.9	27.3	0.52	46.3	46	0 to 100
SPMS	362	67.0	20.0	1.05	67.5	28	10 to 100
DKMS	672	49.1	26.5	1.02	51.9	45	0 to 100
**MSIS-29-PSYCH score (raw):**							
All	4558	24.8	8.9	0.13	24.0	14	9 to 45
PPMS	659	24.7	8.6	0.33	24.0	13	9 to 45
RRMS	2769	24.7	9.0	0.17	24.0	14	9 to 45
SPMS	362	26.1	8.7	0.46	25.0	13	9 to 45
DKMS	672	24.4	9.3	0.36	24.0	14	9 to 45
**MSIS-29-PSYCH score (%):**							
All	4558	43.8	24.8	0.37	41.7	39	0 to 100
PPMS	659	43.5	23.9	0.93	41.7	36	0 to 100
RRMS	2769	43.5	24.9	0.47	41.7	39	0 to 100
SPMS	362	47.5	24.1	1.27	44.4	36	0 to 100
DKMS	672	42.9	25.7	1.00	41.7	39	0 to 100

Descriptions of the variables are shown. Slight differences in totals within categories compared to all are due to the small percentages (<2%) of participants for whom either age, gender or type of MS was missing.

### Characteristics of respondents

There were 28.7% men and 70.9% women in the sample and 0.4% did not record their gender (N = 4558). The types of MS were: 14.8% primary progressive MS (PPMS), 62.1% relapsing-remitting MS (RRMS), 8.1% secondary progressive MS (SPMS) and 15.1% did not know their type of MS (DKMS) (N = 4462). The mean age of the respondents was 50.7 years (SE 0.17, SD 11.2) with a median of 51 years (IQR 16). The mean time since diagnosis (by a neurologist) was 10.9 years (SE 0.15, SD 8.9) with a median of 9 years (IQR 12). The mean raw MSIS-29-PHYS score was 60.5 (SE 0.32, SD 21.6) which equates to 50.6%, with a median of 62 (IQR 35). The mean raw MSIS-29-PSYCH score was 24.8 (SE 0.13, SD 8.9) which equates to 43.8%, with a median of 24 (IQR 14). Fuller details are given in Table 1, including values by gender and type of MS.

### Relationships between continuous variables

There was a weak positive relationship between MSIS-29-PHYS and age (*rho* = 0.21, *p*<0.001) and a similar relationship with time since diagnosis (*rho* = 0.24, *p*<0.001). However, there was a weak negative relationship between MSIS-29-PSYCH and age (*rho* = −0.10, *p*<0.001) and little or no relationship with time since diagnosis (*rho* = −0.03, not sig). This confirms the guidance to use the scales separately and not combined as a single score, as otherwise differential effects on physical and psychological well-being could be masked [2].

### Frequencies of physical and psychological impact

**Figure 1:**
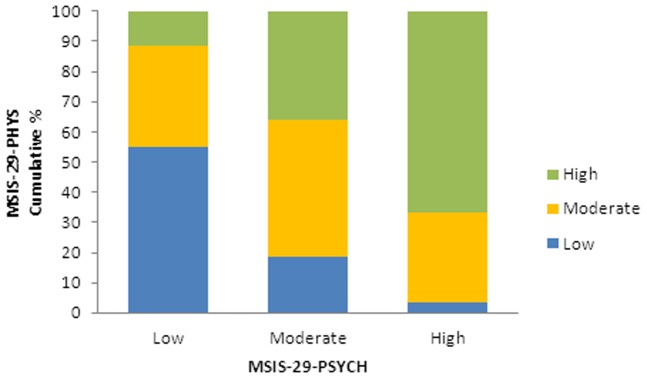
Proportions of respondents with low moderate and high MSIS-29-PHYS and MSIS-29-PSYCH scores. The scores were divided into tertiles: low moderate and high, and the proportions of respondents in each tertile for MSIS-29-PHYS and MSIS-29-PSYCH were compared.

The physical and psychological scales were divided into tertiles to represent low, moderate and high scores in order to study frequencies. There were just over 30% of people in each tertile for MSIS-29-PHYS, and approximately 40% with a low score, 40% with a moderate score and 19% with a high score for MSIS-29-PSYCH (Table 2). The proportions of people in each tertile were assessed based on their type of MS. For MSIS-29-PHYS it was found that the largest proportion of people with PPMS were in the high tertile (45.2%), and similarly for those with SPMS (51.7%), whereas for the people with RRMS, the largest group was in the low tertile (37.4%). The proportion of people who did not know their type of MS (DKMS) was greatest in the moderate tertile (37.4%) with almost equal proportions in the low and high tertiles (Table 2) (chi squared *p*<0.001). The patterns within the tertiles for MSIS-29-PSYCH were fairly similar among the MS types, but with slightly higher proportions of people with SPMS in the moderate and high tertiles (Table 2), however this was not statistically significant. The relationship between the scales was again shown by comparing the low, moderate and high tertiles of the MSIS-29-PHYS and MSIS-29-PSYCH scores. It can be seen that 55.0% of respondents who have a low MSIS-29-PHYS score also have a low MSIS-29-PSYCH score, 45.6% with a moderate MSIS-29-PHYS score have a moderate MSIS-29-PSYCH score, and 66.6% of the people with a high MSIS-29-PHYS score also fall into the high tertile on MSIS-29-PSYCH. The converse was also evident with notably smaller proportions of respondents having a low score on one scale and a high score on the other (Figure 1) (chi squared *p*<0.001).

**Table 2:** Frequencies of low, moderate and high MSIS-29 scores among the cohort.

Category	MSIS-29-PHYS			
	Low	Moderate	High	Total
	(Lower tertile)	(Middle tertile)	(Upper tertile)	
All: No. (in each tertile)	1380	1720	1458	4558
% (in each tertile)	30.3	37.7	32.0	100.0
**Type of MS**				
PPMS: No.	87	274	298	659
%	13.2	41.6	45.2	100.0
RRMS: No.	1035	1000	734	2769
%	37.4	36.1	26.5	100.0
SPMS: No.	21	154	187	362
%	5.8	42.5	51.7	100.0
DKMS: No.	212	251	209	672
%	31.5	37.4	31.1	100.0
	**MSIS-29-PSYCH**			
All: No.	1634	1840	884	4558
%	40.2	40.4	19.4	100.0
**Type of MS**				
PPMS: No.	266	266	127	659
%	40.4	40.4	19.2	100.0
RRMS: No.	1127	1106	536	2769
%	40.7	39.9	19.4	100.0
SPMS: No.	125	161	76	362
%	34.5	44.5	21.0	100.0
DKMS: No.	278	267	127	672
%	41.4	39.7	18.9	100.0
	**MSIS-29-PHYS**			
**MSIS-29-PSYCH**				
Low: No.	1009	614	211	1834
%	55.0	33.5	11.5	100.0
Moderate: No.	342	840	658	1840
%	18.6	45.6	35.8	100.0
High: No.	29	266	589	884
%	3.3	30.1	66.6	100.0

### Physical and psychological impact by MS type

**Figure 2:**
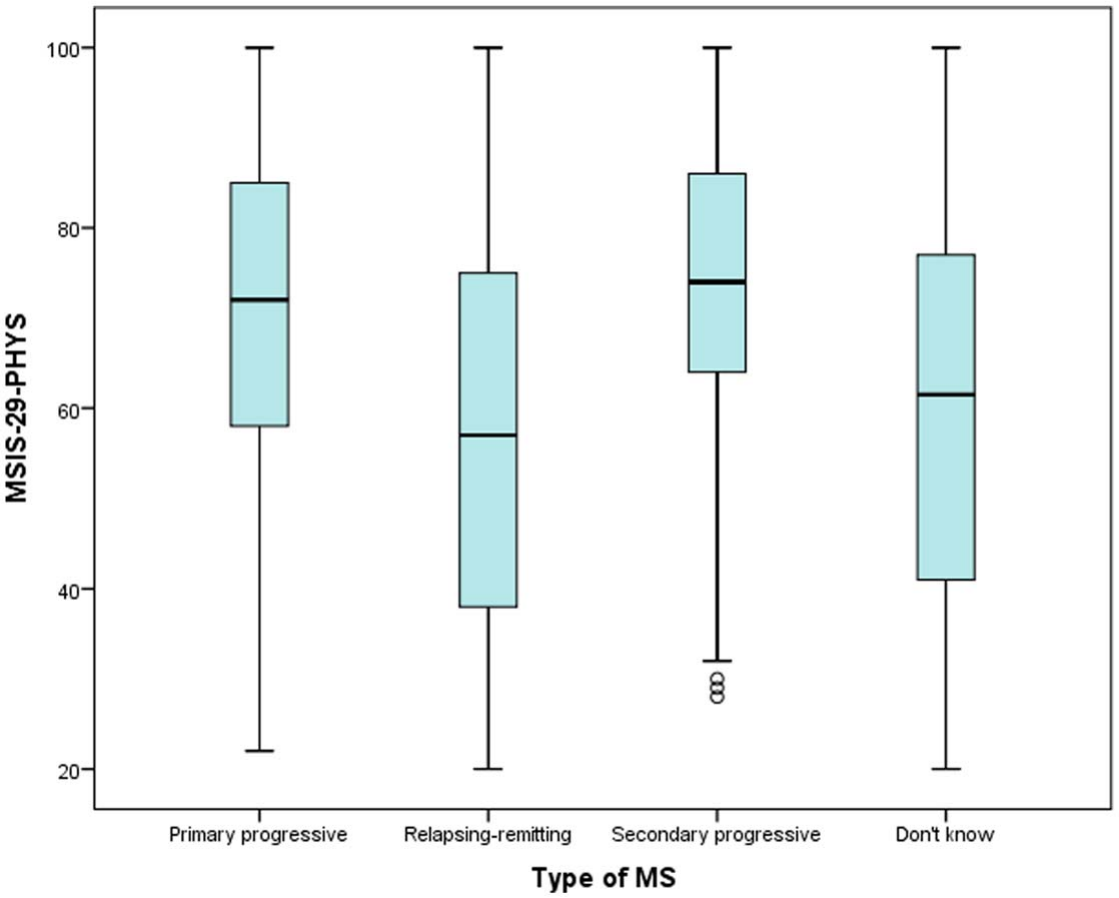
The MSIS-29-PHYS scores by type of MS. The relative MSIS-29-PHYS scores of people who report having different types of MS are indicated.

**Figure 3:**
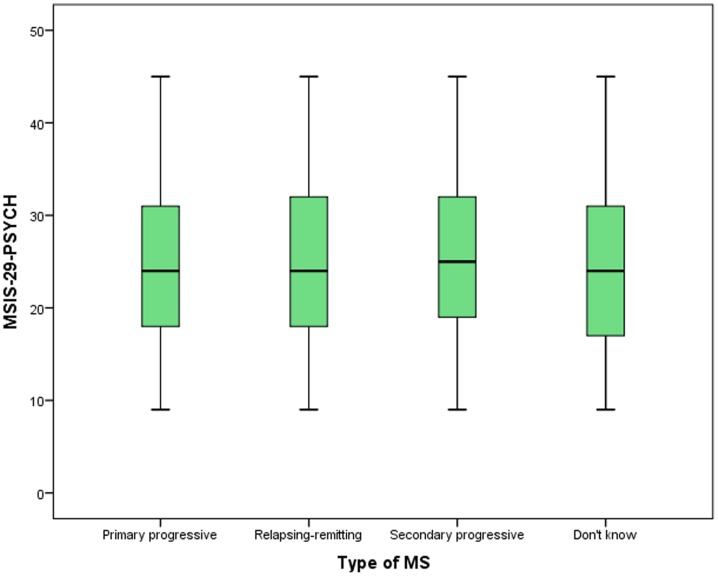
The MSIS-29-PSYCH scores by type of MS. The relative MSIS-29-PSYCH scores of people who report having different types of MS are indicated.

Kruskall-Wallis tests were used to examine how the MSIS-29-PHYS and MSIS-29-PSYCH scores varied by type of MS. It was found that MSIS-29-PHYS scores were higher in progressive types of MS compared to RRMS, and were highest in SPMS (*p*<0.001, *N* = 4462). MSIS-29-PSYCH scores were also highest in SPMS (*p* = 0.03, *N* = 4462). The measures of spread and central tendency are shown in Table 1 and the patterns are illustrated in Figures 2 and 3.

### Impact on the potential workforce

**Figure 4:**
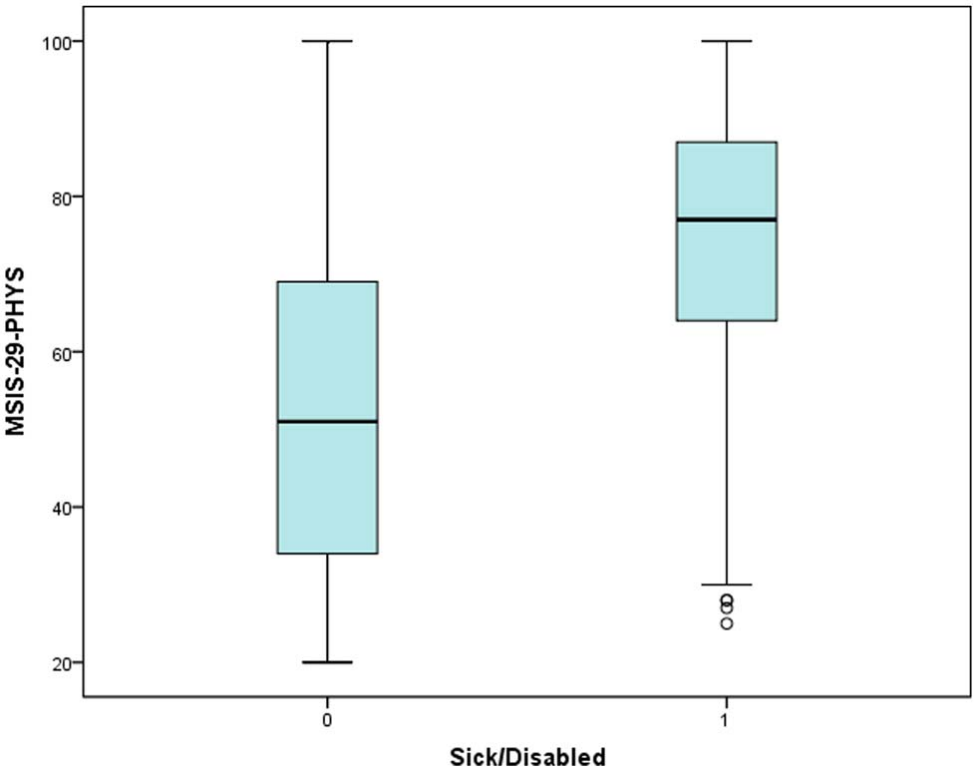
The MSIS-29-PHYS scores for people of workforce age. The scores of people who report they are sick/disabled ( = 1) were compared with those who are not sick/disabled ( = 0).

**Figure 5:**
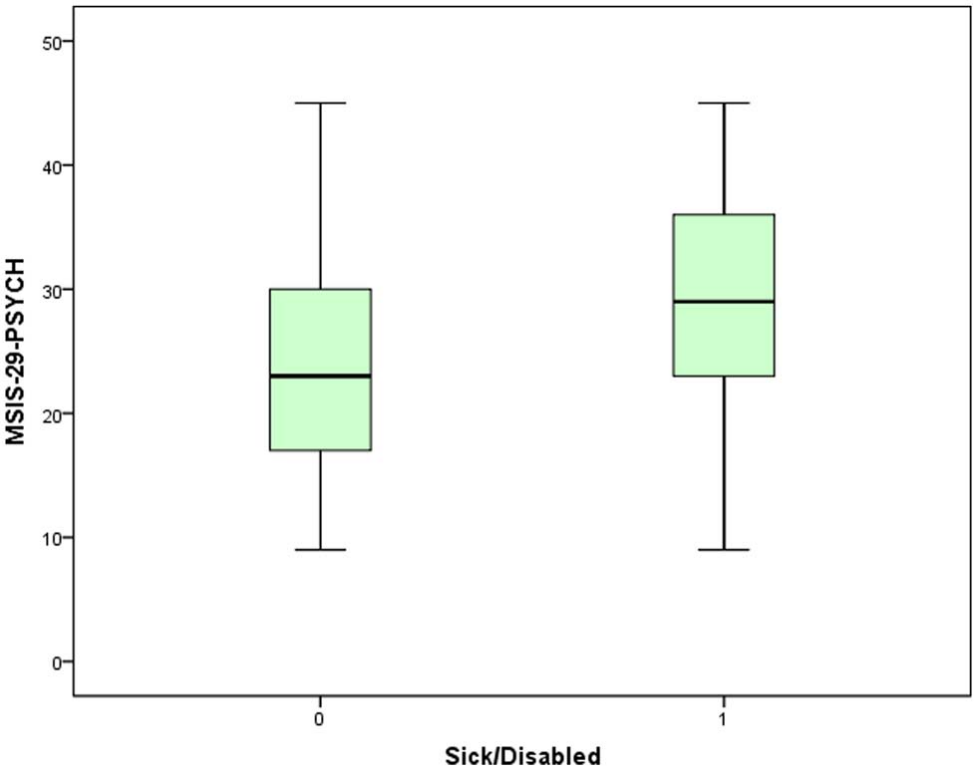
The MSIS-29-PSYCH scores for people of workforce age. The scores of people who report they are sick/disabled ( = 1) were compared with those who are not sick/disabled ( = 0).

The potential workforce was taken as men of ages < = 64 years and women < = 59 years [19]. The respondents provided information on their employment status. Of the potential workforce (N = 3538), 47.6% were in paid employment, 31.3% were sick/disabled, 9.8% were retired, 4.4% were looking after family, 3.6% were unemployed, and the remainder were divided among voluntary work (1.2%) in education (0.6%) or gave other/not applicable as their response (1.5%). By comparison, 76.9% of the general UK potential workforce) are economically active, and 24.5% are recorded as being sick/disabled [19]. The mean MSIS-29-PHYS raw score for the people who stated they were sick/disabled was 75.0 (SE 0.48, SD 16.0, median 77) equating to 68.7%; for the rest of the potential workforce it was 52.3 (SE 0.42, SD 20.7, median 51) equating to 40.4% and, using a Mann Whitney U test, this difference was found to be statistically significant (*p*<0.001, *N* = 3538). MSIS-29-PSYCH scores also differed between those who were sick/disabled and those who were not. The mean MSIS-29-PSYCH raw score for the sick and disabled was 29.1 (SE 0.26, SD 8.5, median 29) equating to 55.7% and for the rest of the potential workforce it was 23.6 (SE 0.18, SD 8.7, median 23) equating to 40.5% (*p*<0.001, *N* = 3538). These results are shown in Figures 4 and 5.

**Figure 6:**
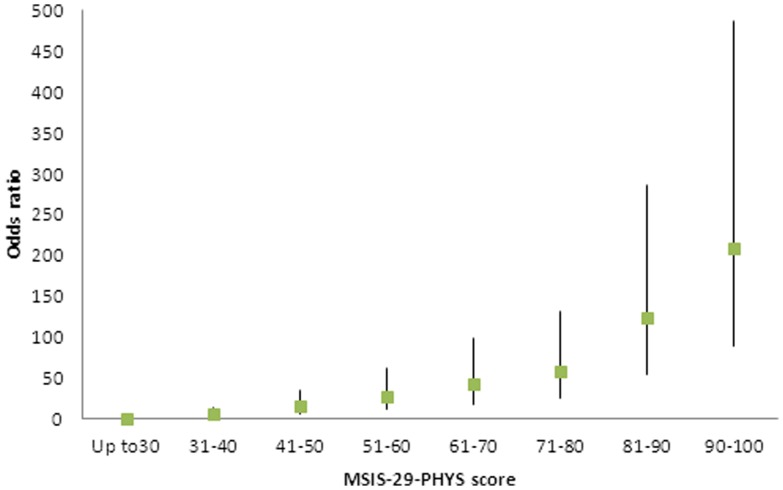
Odds ratios of the workforce being sick/disabled. Logistic regression was conducted to predict the odds of members of the workforce being sick/disabled based on their MSIS-29-PHYS scores in bands of 10 (above the minimum score of 20), relative to the lowest band (up 30). The upper and lower confidence intervals are indicated by vertical lines.

Logistic regression was used to assess how predictive of sick/disabled status the MSIS-29-PHYS and MSIS-29-PSYCH scores were. This was conducted firstly using the tertiles of low, moderate and high MSIS-29-PHYS scores. It was found that, relative to the low tertile, people with a moderate MSIS-29-PHYS score had odds of 7.2 (CI 5.5, 9.4, *p*<0.001) of being sick/disabled, and for the people with a high score the odds were 22.3 (CI 17.0, 29.3, *p*<0.001). Using the same method for the MSIS-29-PSYCH tertiles the values were 2.3 (CI 2.0, 2.8, *p*<0.001) and 4.3 (CI 3.5, 5.2, *p*<0.001), respectively. Since there was a large difference in the means of the MSIS-29-PHYS scores, and the odds ratios were relatively high, further logistic regression was carried out using the raw MSIS-29-PHYS scores in bands of 10 (above the minimum score of 20). The resulting odds ratios (relative to the lowest band of scores up to 30) were plotted against the bands and shown in Figure 6. Confidence intervals are also shown and all *p* values were *p*<0.001. It can be seen that there is a clear rise in the odds of being sick/disabled as the scores increase.

There may be many reasons why a person is or is not actually engaged in gainful employment, but it was found that the MSIS-29-PHYS and MSIS-29-PSYCH scores were significantly lower among those in work compared to the remainder of the cohort. The MSIS-29-PHYS scores of people in work showed a mean of 48.2 (SE 0.48, SD 19.7, median 46) (35.3%) compared to 69.5 (SE 0.44, SD 18.9, median 72) (61.9%) for those not in paid work. For the MSIS-29-PSYCH the values were 22.8 (SE 0.21, SD 8.6, median 22) (38.4%) and 27.5 (SE 0.21, SD 8.8, median 27) (51.4) respectively. For both scales, Mann Whitney U tests showed that the differences were significant (*p*<0.001, *p*<0.001, N = 3538). Logistic regression showed that people with a moderate MSIS-29-PHYS score had odds of 0.25 (CI 0.21, 0.30, p<0.001) of being in paid work, and for the people with a high score the odds were 0.08 (CI 0.06, 0.10, p<0.001), relative to those in the low tertile. Similarly, for the MSIS-29-PSYCH tertiles the values were 0.58 (CI 0.50, 0.67, p<0.001) and 0.29 (CI 0.24, 0.35, p<0.001), respectively. These values equate to 4.0 and 12.5 times the odds of not being in paid work for people with moderate and high MSIS-29-PHYS scores compared to those with low scores, and 1.7 and 3.5 times the odds for people with moderate and high MSIS-29-PSYCH scores, respectively.

## Discussion

### Main findings

This study has examined the physical and psychological impact of MS using >4500 responses to the MSIS-29 via the web portal of the UK MS Register. This scale has been found to be reliable and valid for use in research and clinical settings and our findings using data collected via a web portal are consistent with the reported properties of the scale [2], [4], [5], [7]. This is encouraging and supports the feasibility of using this method as an alternative mode of data collection to collect patient reported outcome data, such as these, in research studies and clinical trials [12]. Our cohort displayed a broad range of ages and MS durations, and was reasonably representative of many of the characteristics of other prevalent MS cohorts [20]–[23]. The mean MSIS-29-PHYS and MSIS-29-PSYCH scores were similar to those reported elsewhere (56.0% and 45.5% respectively, *N* = 703) [2]. Since the MSIS-29-PHYS scale runs from 20 to 100, and MSIS-29-PSYCH from 9 to 45, we have reported the raw scores and percentages based on transformed data for ease of reference. There is some evidence to indicate that the MSIS-29-PHYS and MSIS-29-PSYCH scales are measuring related but distinct constructs and so the use of a combined score was not recommended as it may mask important differential effects on physical and psychological health [2]. Our results concur with this as it was found that MSIS-29-PHYS scores were positively correlated with age, whereas there was a negative relationship between MSIS-29-PSYCH scores and age. Furthermore in our previous study using responses to the HADS, we found a weak negative relationship between age and anxiety, and differences in depression levels among different age groups [16].

There was a moderately strong positive relationship between the MSIS-29-PHYS and MSIS-29-PSYCH, and an examination of frequencies within tertiles showed people with a low MSIS-29-PHYS score tended to have a low MSIS-29-PSYCH score. This was also true for people with moderate scores, and of the people with high MSIS-29-PHYS scores, two-thirds also had high MSIS-29-PSYCH scores. The converse was also true with only low proportions of people having a low or a high score on both scales. These findings further support the relationship between the scales and the importance of jointly, but separately, considering physical and psychological impacts on people with MS. There were differences in the MSIS-29-PHYS and MSIS-29-PSYCH scores depending on the type of MS: people with PPMS or SPMS had higher MSIS-29-PHYS scores than people with RRMS, and people with SPMS had the highest MSIS-29-PSYCH scores. This would be expected due to the variable disease course in different types of MS.

During their validation of the scale, Hobart and colleagues found that people who had retired (N = 390) because of their MS had higher mean MSIS-29-PHYS and MSIS-29-PSYCH scores than those who were in employment (N = 107) [2]. With a much larger sample, we have been able to study the impact of MS on the potential workforce in greater depth, including how predictive the scores are of a person being unable to work. We showed that a greater proportion of people with MS report that they are sick/disabled compared to the general UK workforce, and fewer are in gainful employment [19]. Furthermore, the MSIS-29-PHYS and MSIS-29-PSYCH scores for the sick/disabled group were significantly higher than the remainder of the cohort, and people with higher scores had higher odds of having a status of sick/disabled. This relationship was demonstrated with MSIS-29-PHYS and MSIS-29-PSYCH in tertiles and, since MSIS-29-PHYS scores were the stronger predictor of sick/disability status, it was also shown for this scale divided into bands of 10 and clear incremental increases in the likelihood of people reporting sick/disabled as their employment status were observed. This may be useful to clinicians in assessing people's likely opportunities to be in employment, and we suggest this also reasonably represents a proxy for assessing impact on engaging in general life activities. We chose to use the relationship between MSIS-29 scores and being sick/disabled to assess impact, rather than between MSIS-29 scores and being in paid work, as there may be various reasons why a person may or may not actually be in employment. These include personal circumstances, choices and needs, and the unavailability of work. Nevertheless, we found that the MSIS-29-PHYS and MSIS-29-PSYCH scores were significantly lower among those in paid work compared to the remainder of the cohort, and people with moderate or high MSIS-29-PHYS and MSIS-29-PSYCH scores showed significantly decreased odds of being in paid work. As would be expected, the effects are not as large as found using sick/disabled status as the predicted outcome. But this indicates not only that disability due to MS (as measured by MSIS-29 scores) is strongly predictive of being sick/disabled, but it is also a major factor in why people are not actually taking an active part in the workforce.

### What this study adds

This is the largest known study to look at the impact of MS via the MSIS-29, and as such it may be of value for comparative purposes in studies where large numbers are not accessible. Studies using the scale in different settings were recommended by Hobart and colleagues [2], and by comparing the internal properties of the scale with published findings, we have shown that it can be used to collect data for research via an internet-based web portal. This study has provided new information on disability in MS directly from the people with the condition, and it has shown how physical and psychological disability levels vary with type of MS and with age and disease duration. The findings examining MSIS-29-PHYS and MSIS-29-PSYCH scores against age confirmed the suggested guidance provided by Hobart and colleagues [2] that the MSIS-29 should be used as physical and psychological sub-scales, not as a combined score. We found that increasing disability scores, especially on the physical scale, are strongly predictive of the potential workforce having an employment status of sick/disabled. We have also shown the increasing odds of a sick/disabled status based on MSIS-29-PHYS in 10-score bands. This may be useful to clinicians in estimating the impact of MS on people of workforce age, and may also be used to represent a proxy measure for gauging the likely impact of MS on life in general.

### Limitations

This study was based on self-reported information and it is possible that the respondents were not necessarily a fully representative sample of people with MS in the UK. It has been reported that data may be skewed when using web-based data collection methods, as the technology may pose a barrier to the elderly, disadvantaged, technically inexperienced or cognitively impaired [24], [25].However, we have found that the education profile our cohort is similar to the UK population, and many of their MS characteristics are typical of other MS prevalent populations, but our proportion of people with SPMS is lower than might be expected [11] The reasons for this are not known at this stage, but they may be partly because half to two-thirds of people initially diagnosed with RRMS go on to develop SPMS within 10 to 15 years, and some of the respondents may be reporting an earlier diagnosis [23], [26]. We will be able to estimate and address any response bias in portal data using the linkable data from clinical sites and routine sources, as these data accrue. The MSIS-29 scale has been shown to be useful in measuring the impact of MS, but we have observed some limitations. For example, unlike the HADS, it cannot clearly distinguish between depression and anxiety, and as such, its psychological component appears to be of more limited value in guiding clinical care. The UK MS Register is still in its early stages, with further work underway. Although many studies will be made possible as the data increase over time, others will still require more traditional settings and data collection methods.

### Future work

We have a programme of further work underway and plan to analyse the other questionnaires delivered via the platform. These will include the EQ-5D which is used to assess health status for clinical and economic evaluation [15], and questionnaires collecting information on medication records and symptoms. We plan to re-examine the MSIS-29 in conjunction with reported symptoms and medication records to determine their patterns in relation to disability scores. We have recently updated to version 2 of the MSIS-29, including the walking scale and, as data accrue, we will be able to compare responses to the two versions of the scale. We are also carrying out qualitative research to ensure that the Register meets the needs of people with MS [27], [28]. As Register data continue to be collected, we will link self-reported information with clinical and administrative data to compare information between the data sources and to enable more in-depth studies, only possible via data linkage at the individual level.

### Conclusion

It is well-known that MS results in numerous, varied disabling effects and has a major impact on many aspects of life. The MSIS-29 has been proven to be a reliable and valuable tool for use in MS care [5], [9] and the findings of this novel large-scale study, with data collection via an internet-based questionnaire delivery platform, are consistent with this. Our findings support the use of the MSIS-29 as two sub-scales, one for physical effects and one for psychological effects, not as a combined score. MSIS-29 scores can be used to gauge the impact of MS on the potential workforce: the physical and psychological scores can be used to predict the odds of people having an employment status of sick/disabled, with MSIS-29-PHYS scores showing the strongest effect. Our results underline the importance of using a reliable disease-specific scale such as the MSIS-29 for regular monitoring of people with MS to help them take an active part in the workforce and in the enjoyment of general life activities.
